# In-Hospital Outcomes of Patients With Acute Respiratory Distress Syndrome Treated With Extracorporeal Membrane Oxygenation

**DOI:** 10.7759/cureus.68745

**Published:** 2024-09-05

**Authors:** Abdul Rasheed Bahar, Yasemin Bahar, Chaitu Dandu, Mohamed S Alrayyashi, Mohamed Zghouzi, Adam Chalek, M. Chadi Alraies

**Affiliations:** 1 Internal Medicine, Wayne State University Detroit Medical Center, Detroit, USA; 2 Internal Medicine, Wayne State University, Detroit, USA; 3 Vascular Surgery, Wayne State University School of Medicine, Detroit, USA; 4 Internal Medicine, Wayne State University School of Medicine, Detroit, USA; 5 Cardiology, Wayne State University Detroit Medical Center, Detroit, USA

**Keywords:** ards, ecmo, in-hospital outcomes, inpatient mortality, vv ecmo

## Abstract

Background: Treatment of acute respiratory distress syndrome (ARDS) with extracorporeal membrane oxygenation (ECMO) remains controversial.

Objective: This study aims to examine outcomes in ARDS patients treated with or without ECMO.

Methods: Using the National Inpatient Sample (NIS) database, all ARDS patients including those who were treated with ECMO were included in the analysis. Univariable and multivariable logistic regressions were used to estimate the odds of in-hospital outcomes between groups.

Results: A total of 2,540,350 patients were identified (2,538,849 with ARDS; 1,501 with ARDS on ECMO). The patients who underwent ECMO included younger patients and more men. Using ECMO in ARDS patients was associated with higher in-hospital mortality, cardiopulmonary arrest, major bleeding, sepsis, acute kidney injury, and longer hospital stays (31.7 vs. 8.3 days; p < 0.001 for all). A subgroup analysis based on age and sex had similar outcomes.

Conclusion: Using ECMO in patients with ARDS was associated with worse in-hospital outcomes, including mortality and length of stay.

## Introduction

Acute respiratory distress syndrome (ARDS) is a life-threatening condition that affects nearly 190,000 individuals in the USA yearly [1]. In ARDS, the severe and rapid widespread inflammation leads to fluid accumulation in the lungs, which impairs gas exchange resulting in life-threatening hypoxemia and severe respiratory distress [2]. In severe cases, ARDS can be associated with a mortality rate exceeding 60% [3]. During the COVID-19 pandemic, there was a sharp increase in the occurrence of ARDS amongst patients due to the widespread SARS-COV-2 virus. Furthermore, it has been shown that COVID-19-related ARDS is associated with a higher mortality rate compared to ARDS from other causes (26-61.5% vs. 35.3-40%) [4]. Therefore, the medical and scientific community has started to pay more attention to the treatment of the disease given its high mortality. Extracorporeal membrane oxygenation (ECMO) is an extracorporeal technique that helps provide circulatory and respiratory support to patients with hypoxemic and hypercapnic respiratory failure. ECMO involves a system where blood is drawn from the venous vascular system through a catheter, circulated by an external pump, and then reintroduced into either the venous or arterial system to support circulation in the body [5]. There are two basic types of ECMO, venovenous (VV) ECMO, which provides respiratory support only, and venoarterial (VA) ECMO, which bypasses the heart and lungs [6]. Traditionally, ECMO has been used in patients who require additional support on maximum mechanical ventilation and cardiac mechanical support. The usage of ECMO in patients with ARDS has increased during the COVID-19 pandemic [7]. Studies exploring the use of ECMO in patients with severe ARDS have shown conflicting results. A meta-analysis in 2020 showed significantly reduced 90-day mortality in severe ARDS patients with early uses of ECMO compared to conventional ventilatory support [8]. The EOLIA trial showed that early use of ECMO did not significantly improve mortality at 60 days in patients with severe ARDS. Still, when used as a rescue modality, ECMO might help improve survival [9]. The current data about the ECMO roles and mortality benefits are limited and unclear. There is a lack of literature comparing the outcome of ARDS on ECMO. In this study, we aimed to assess the in-hospital outcomes in patients with ARDS with and without ECMO support.

## Materials and methods

Data source

This real-world population-based study was retrospectively analyzed from the National Inpatient Sample (NIS) database. The NIS is the largest publicly available all-payer inpatient healthcare database in the United States, designed to provide nationally representative estimates of inpatient utilization, access, cost, quality, and outcomes. The NIS database allows for the national assessment of hospital discharges among patients of different age groups across all payer types from all US hospitals [10]. The included data are completely de-identified and hence exempted from approval by the institutional review board. NIS contains data from almost 20 million discharges each year, representing more than 100 million weighted discharges of national estimates. NIS is managed and closely mandated by the Agency for Healthcare Research and Quality (AHRQ) [10]. The study was conducted at Wayne State University School of Medicine over a duration of approximately two months. Since the NIS is a publicly available, anonymized national database with prior ethical approval from the AHRQ, our study was exempt from Institutional Review Board (IRB) approval.

Our study adheres to the methodological design checklist proposed by Khera et al. (2017) to mitigate common study design errors in NIS-based research [11].

Study population and variables

We utilized nationally weighted 2002-2017 NIS claims to select all US adult patients (>18 years) with a diagnosis of ARDS. The included sample was divided into two groups: patients requiring ECMO and those without ECMO support. The International Classification of Diseases (ICD) Clinical Modification (ICD-CM) codes were used to identify populations of interest. ICD-9 and ICD-10 codes for the ECMO procedure used in the analysis were 39.65 and 5A1522G, respectively. The ICD-CM codes were validated through an independent review by two authors with minimal interobserver variability (<5%), and discrepancies were resolved through consultation with a third author and further code iteration as needed. The list of ICD-9/10-CM diagnoses and procedural codes used in the study are presented in the appendix (Table S1).

The primary outcome was all-cause in-hospital mortality. Secondary outcomes included non-fatal stroke, and post-transplant complications, procedure-related bleeding, major bleeding, acute kidney injury (AKI), pneumonia, sepsis, length of stay (LOS), and cost of hospitalization. The patient demographics and baseline comorbidities were abstracted for both groups. The data included age, gender, race, hospital data, hyperlipidemia, hypertension, diabetes mellitus, cerebrovascular disease, peripheral vascular disease, chronic kidney disease without dialysis, chronic obstructive pulmonary disorder, and other comorbidities tracked in the NIS database using the Charlson method for documentation [12]. LOS, total hospitalization charges, and patient demographics were directly obtained from the NIS database. Total hospital charges reflect the amount a patient was billed for the entire hospital stay, but they do not reflect the actual cost of care. The Healthcare Cost and Utilization Project (HCUP) provides data with hospital-specific cost-to-charge ratios based on inpatient costs across all payers [13]. Using this information, total hospital costs were calculated by multiplying the hospital charges by the corresponding cost-to-charge ratio. Patient demographics included age (in years), sex, race (categorized as Caucasian, Black, Hispanic, Asian or Pacific Islander, Native American, and other), primary expected payer (Medicare, Medicaid, private insurance, and uninsured), hospital bed size (small, medium, and large), teaching status, hospital region (Northeast, Midwest, South, and West), and urban location [14].

Statistical analysis

All analyses were conducted using weighted NIS data in compliance with the HCUP recommendations [15]. Each hospital admission was linked to a discharge weight used to calculate projected national estimates for in-hospital outcomes. This approach accounted for clustering, weighting, and stratification to ensure that the results were representative of the broader US population. Missing data were assessed and treated using multiple imputations by predictive mean matching. This method preserves the distribution and variability of the observed data while incorporating the relationships among variables [16,17].

Patient demographics and baseline comorbidities between the comparison groups were reported using descriptive statistics: chi-square (x2) and means for categorical and normally distributed continuous variables, respectively. For non-normally distributed data, median and interquartile percentiles (IQR) were reported. The in-hospital outcomes for dichotomous variables were compared using the Cochran-Mantel-Haenszel (CMH) test to calculate unadjusted odds ratios (uOR). The mean and standard deviation (SD) of normally distributed continuous variables (LOS and cost) were compared using independent t-test analysis. The mean ranks for non-normally distributed data were compared with the Mann-Whitney U test. To address the impact of potential confounders, differences in the in-hospital outcomes were assessed using risk-adjusted logistic regression. Multivariable logistic regression was risk-adjusted for age, gender, race, hospital characteristics, and baseline comorbidities. The list of potential confounders accounted for regression analysis is presented in Table S2. The allowable tolerance in the propensity score for the underlying logistic regression model was set at 0.02 unit difference in odds of “between neighboring pairs”. Variables with cell sizes <10 were excluded given NIS reporting guidelines. All pooled estimates were presented with 95% confidence intervals (CIs) and an alpha criterion of P < 0.05 was regarded as statistically significant. The analyses were computed using SPSS version 24.0 (IBM Corp., Armonk, NY) and R statistics version 3.3 (R Foundation for Statistical Computing, Vienna, Austria).

## Results

Study population characteristics

A total of 2,540,350 patients who had a diagnosis of ARDS were included in the study. Of these, 2,538,849 (99.5%) were ARDS patients not placed on ECMO, and 1501 (0.05%) were ARDS patients placed on ECMO. The baseline characteristics of both groups are summarized in Table 1.

**Table 1:** Baseline characteristics of patients with ARDS and patients with ARDS on ECMO. Values are expressed as mean ± SD for continuous variables or percentages for categorical variables. ARDS: acute respiratory distress syndrome; ECMO: extracorporeal membrane oxygenation; PUD: peptic ulcer disease; PAD: peripheral artery disease; FHCAD: family history of premature coronary artery disease; OSA: obstructive sleep apnea; LD: liver disease; PCI: percutaneous coronary intervention; CABG: coronary artery bypass graft; HF: heart failure; MI: myocardial infarction; AS: aortic stenosis; MS: mitral stenosis; CAD: coronary artery disease; ESRD: end-stage renal disease; DM: diabetes mellitus. Transfer out indicator: 0 – not a transfer; 1 – transferred out to a different acute care hospital; 2 – transferred out to another type of health facility. Transfer in indicator: 0 – not transferred in; 1 – transferred in from a different acute care hospital; 2 – transferred in from another type of health facility.

Variables	ARDS = 2,538,849 (99.95%)	ARDS on ECMO = 1501 (0.05%)
Age (years)	53.2 ± 29.54	32.20 ± 22.49
Female gender	1,290,382 (50.9%)	661 (44.1%)
Race		
White	1,381,361 (54.4%)	678 (45.2%)
Black	338,437 (13.3%)	228 (15.2%)
Hispanic	287,069 (11.3%)	178 (11.9%)
Asian or Pacific Islander	63,249 (2.5%)	59 (4%)
Native American	16,165 (0.6%)	31 (2.1%)
Others	71,488 (2.8%)	108 (7.2%)
Length of stay (days)	8.29 ± 11.3	31.69 ± 48.11
Total charges (US$)	$72,340 ± 157,074	$667,840 ± 801,916
Elective hospitalization	2,254,085 (88.78%)	1,256 (83.7%)
Admission on weekend	609,661 (24%)	297 (19.8%)
Transfer in indicators	N = 341,091	N = 540
0	282,430 (82.8%)	230 (42.6%)
1	45,800 (13.4%)	290 (53.7%)
2	12,860 (3.8%)	20 (3.7%)
Transfer out indicators	N = 342,940	N = 550
0	252,760 (73.7%)	340 (61.8%)
1	22,470 (6.6%)	110 (20%)
2	67,710 (19.8%)	100 (18.2%)
Bed size	N = 2,189,114	N = 940
Small	312,853 (14.3%)	109 (11.6%)
Medium	552,173 (25.2%)	243 (25.9%)
Large	1,324,088 (60.5%)	588 (62.6%)
Location/teaching status	N = 2,189,115	N = 940
Rural	266,213 (12.2%)	0 (0.0%)
Urban non-teaching	701,957 (32.1%)	10 (1.1%)
Urban teaching	1,220,945 (55.8%)	930 (98.9%)
Hospital region	N = 2,194,660	N = 951
Northeast	511,909 (23.3%)	411 (43.2%)
Midwest	467,657 (21.3%)	230 (24.1%)
South	772,530 (35.2%)	190 (20.0%)
West	442,564 (20.2%)	120 (12.7%)
Comorbidities	N = 2,538,849	N = 1501
PUD	1,061 (0.042%)	0 (0.0%)
Pulmonary disease	256,424 (10.1%)	50 (3.3%)
Pulmonary circulation disorders	157,408 (6.2%)	126 (8.4%)
Alcohol use	33,005 (1.3%)	20 (1.3%)
Anemia	73,627 (2.9%)	50 (3.3%)
Electrolytes abnormalities	901,291 (35.5%)	922 (61.4%)
Hypothyroidism	205,647 (8.1%)	40 (2.7%)
Weight loss	58,394 (2.3%)	30 (2.0%)
Obesity	129,481 (5.1%)	130 (8.7%)
Coagulopathy	66,010 (2.6%)	280 (18.7%)
PAD	66,010 (2.6%)	9 (0.6%)
FHCAD	30,466 (1.2%)	10 (0.7%)
OSA	101,554 (4%)	30 (2%)
Liver disease	101,554 (4%)	157 (10.5%)
Drug use	215,802 (8.5%)	59 (3.9%)
Smoking	368,133 (14.5%)	189 (12.6%)
Prior PCI	45,699 (1.8%)	0 (0.0%)
Prior CABG	78,704 (3.1%)	10 (0.7%)
HF	682,950 (26.9%)	398 (26.5%)
Prior MI	86,321 (3.4%)	30 (2%)
Atrial fibrillation	398,599 (15.7%)	118 (7.9%)
Atrial flutter	45,699 (1.8%)	88 (5.9%)
AS	7,617 (0.3%)	10 (0.7%)
MS	2,539 (0.1%)	9 (0.6%)
CAD	439,221 (17.3%)	120 (8%)
Vascular complications	10,155 (0.4%)	19 (1.3%)
ESRD	129,481 (5.1%)	239 (15.9%)
Dyslipidemia	416,371 (16.4%)	90 (6%)
Hypertension	616,940 (24.3%)	109 (7.3%)
DM	83,782 (3.3%)	100 (6.7%)

The patients with ARDS who were placed on ECMO were significantly younger, with a median age of 32.20 years (interquartile range, IQR: 22.49 years) compared to a median age of 53.2 years (IQR: 29.54 years) in the non-ECMO group. Compared to the non-ECMO group, the majority of patients who required ECMO were male (59.9% vs. 49.1%). The prevalence of pulmonary circulation disorders, obesity, electrolyte abnormalities, coagulopathies, lung disease, aortic stenosis, multiple sclerosis, end-stage renal disease, and diabetes mellitus were more prevalent in the patients with ARDS on ECMO, whereas hypothyroidism, paralysis, depression, peripheral arterial disease, obstructive sleep apnea, drug use, prior coronary artery bypass graft, atrial fibrillation, coronary artery disease, dyslipidemia, and hypertension were all more prevalent in the patients with ARDS not on ECMO. The presence of peptic ulcer disease, alcohol use, weight loss, and family history of premature coronary artery disease (FHCAD) were similar between both groups.

In-hospital outcomes

**Figure 1:**
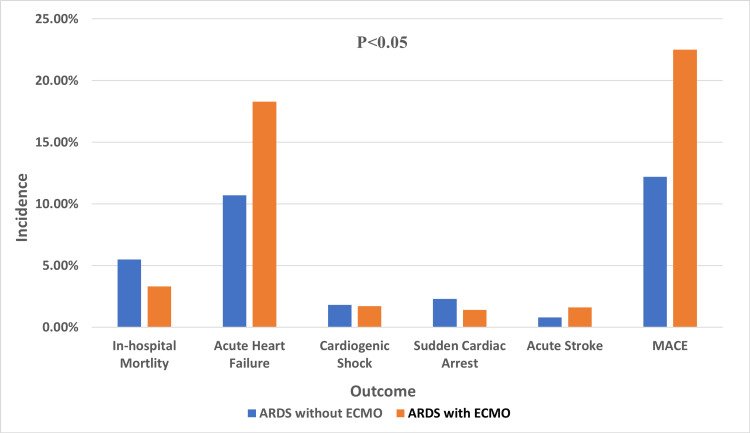
Column chart demonstrating in-hospital outcomes of ARDS patients with and without ECMO treatment. ARDS: acute respiratory distress syndrome; ECMO: extracorporeal membrane oxygenation; MACE: major adverse cardiac events.

Using ECMO for patients with ARDS was associated with higher in-hospital mortality (48.4% vs. 12.8%, adjusted odds ratio (aOR): 12.83; 95% CI: 11.2-14.71, p < 0.001), cardiopulmonary arrest (23% vs. 2%, aOR: 12.69; 95% CI: 10.89-14.7, p < 0.001), major bleeding (24.5% vs. 6.8%, aOR: 5.54; 95% CI: 4.78-6.41, p < 0.001), sepsis (46.9% vs. 15.5%, aOR: 3.88; 95% CI: 3.38-4.46, p < 0.001), and AKI (51.7% vs. 17.3%, aOR: 5.28; 95% CI: 4.58-6.07, p < 0.001). In-hospital outcomes of patients with ARDS treated with ECMO are shown in Figure 1.

However, the odds of stroke were higher in ARDS patients not treated with ECMO (2.4% vs. 1.3%, aOR: 0.55; 95% CI: 0.35-0.86, p = 0.009) (Table 2).

**Table 2:** In-hospital outcomes in patients with ARDS and patients with ARDS on ECMO. The in-hospital outcomes for dichotomous variables were compared using the Cochran–Mantel–Haenszel (CMH) test to calculate unadjusted odds ratios (uOR). Multivariable logistic regression was used to adjust for demographics (age, sex, race), hospital characteristics (region, bed size, teaching status), and all comorbidities listed in Table 1. The odds ratios and 95% confidence intervals (CI) are shown. “Ref.” indicates ARDS as the reference group. ARDS: acute respiratory distress syndrome; ECMO: extracorporeal membrane oxygenation; OR: odds ratio.

	ARDS (N = 2,538,849)	ARDS on ECMO (N = 1501)	p-value
In-hospital mortality			
N (%)	323,549 (12.8%)	727 (48.4%)	
Unadjusted OR (95% CI)	Ref.	6.420 (5.801-7.104)	<0.05
Adjusted OR (95% CI)	Ref.	12.834 (11.202-14.705)	<0.05
Cardiopulmonary arrest			
N (%)	51,553 (2%)	347 (23.1%)	
Unadjusted OR (95% CI)	Ref.	14.499 (12.855-16.353)	<0.05
Adjusted OR (95% CI)	Ref.	12.693 (10.889-14.796)	<0.05
Stroke			
N (%)	60,340 (2.4%)	20 (1.3%)	
Unadjusted OR (95% CI)	Ref.	0.554 (0.357-0.862)	<0.05
Adjusted OR (95% CI	Ref.	0.554 (0.357-0.862)	<0.05
Major bleeding			
N (%)	171,673 (6.8%)	368 (24.5%)	
Unadjusted OR (95% CI)	Ref.	4.476 (3.979-5.053)	<0.05
Adjusted OR (95% CI)	Ref.	5.535 (4.776-6.414)	<0.05
Sepsis			
N (%)	392,307 (15.5%)	704 (46.9%)	
Unadjusted OR (95% CI)	Ref.	4.830 (4.364-5.345)	<0.05
Adjusted OR (95% CI)	Ref.	3.881 (3.380-4.457)	<0.05
Acute kidney injury			
N (%)	439,321 (17.3%)	776 (51.7%)	
Unadjusted OR (95% CI)	Ref.	5.112 (4.619-5.656)	<0.05
Adjusted OR (95% CI)	Ref.	5.275 (4.580-6.074)	<0.05

In addition, the patients treated with ECMO had longer hospital stays (31.7 vs. 8.3 days, p < 0.001) (Table 1).

In a subgroup analysis of patients with ARDS on VV ECMO vs. patients with ARDS not on ECMO, VV ECMO was associated with lower in-hospital mortality (uOR: 0.352, 95% CI: 0.274-0.453, p < 0.001) and lower cardiopulmonary arrest (uOR: 0.287, 95% CI: 0.208-0.397, p< 0.001). On the other hand, patients on VA ECMO showed a higher risk of cardiopulmonary arrest (uOR: 0.2.254, 95% CI: 1.546-3.287, p < 0.001) and a lower risk of major bleeding (uOR: 0.137, 95% CI: 0.071-0.266, p < 0.001) compared to patients not on ECMO. In-hospital outcomes in patients with ARDS on VA ECMO and VV ECMO vs. patients without ECMO are shown in Table 3.

**Table 3:** In-hospital outcomes in patients with ARDS on VA ECMO and patients with ARDS on VV ECMO vs. ARDS without ECMO. The in-hospital outcomes for dichotomous variables were compared using the Cochran–Mantel–Haenszel (CMH) test to calculate unadjusted odds ratios (uOR). Multivariable logistic regression was used to adjust for demographics (age, sex, race), hospital characteristics (region, bed size, teaching status), and all comorbidities listed in Table 1. ARDS: acute respiratory distress syndrome; ECMO: extracorporeal membrane oxygenation; uOR: unadjusted odds ratio; VV: venovenous; VA: venoarterial.

	VA ECMO (n = 150)	p-value	VV ECMO (n = 390)	p-value
In-hospital mortality				
Unadjusted OR (95% CI)	0.907 (0.622-1.322)	0.611	0.352 (0.274-0.453)	<0.05
Cardiopulmonary arrest				
Unadjusted OR (95% CI)	2.254 (1.546-3.287)	<0.001	0.287 (0.208-0.397)	<0.05
Major bleeding				
Unadjusted OR (95% CI)	0.137 (0.071-0.266)	<0.001	1.050 (0.788-1.398)	>0.05
Sepsis				
Unadjusted OR (95% CI)	1.082 (0.743-1.576)	0.683	1.280 (0.989-1.657)	>0.05
Acute kidney injury				
Unadjusted OR (95% CI)	1.674 (1.094-2.562)	0.018	0.986 (0.755-1.287)	>0.05

A subgroup analysis demonstrated that ECMO was associated with increased in-hospital mortality in patients younger than 65 years (47.2% vs. 8.5%, aOR: 9.618; 95% CI: 8.66-10.68, p < 0.001) and older than 65 years (67.4% vs. 18.2%, aOR: 9.273; 95% CI: 5.95-14.45, p < 0.001). ECMO was associated with increased odds of AKI, major bleeding, and sepsis in both age groups (p < 0.001). While ECMO was associated with a higher rate of cardiopulmonary arrest in patients younger than 65 years, it was not statistically significant in patients older than 65 years. Furthermore, ECMO was associated with increased in-hospital mortality in both male (56% vs. 13.3%, aOR: 8.30; 95% CI: 7.24-9.51, p < 0.001) and female (38.8% vs. 12.3%, aOR: 4.53; 95% CI: 3.876-5.30, p < 0.001) patients. Also, ECMO increased the odds of AKI, major bleeding, sepsis, and cardiopulmonary arrest in both female and male patients (p < 0.001). Outcomes of ARDS on ECMO vs. ARDS without ECMO sub-grouped by age and sex are shown in Tables 4, 5.

**Table 4:** In-hospital outcomes of ARDS patients with and without ECMO subgrouped by gender. Multivariable logistic regression was used to adjust for confounding factors such as age, sex, race, hospital characteristics (region, bed size, teaching status), and all comorbidities listed in Table 1. The allowable tolerance for the underlying logistic regression model was set at 0.02 unit difference in odds of “between neighboring pairs.” ARDS: acute respiratory distress syndrome; ECMO: extracorporeal membrane oxygenation; aOR: adjusted odds ratio; AKI: acute kidney injury.

	Male				Female			
Outcomes	ARDS (N = 1,245,630)	ARDS on ECMO (N = 840)	aOR (95%CI)	P-value	ARDS (N = 1,290,382)	ARDS on ECMO (N = 661)	aOR (95%CI)	P-value
AKI	234,178 (18.8%)	497 (59.2%)	6.27 (5.46-7.20)	<0.0001	205,171 (15.9%)	278 (42.1%)	3.84 (3.29-4.49)	<0.05
Major bleed	90,931 (7.3%)	182 (21.7%)	3.50 (2.97-4.12)	<0.0001	80,004 (6.2%)	186 (28.2%)	5.93 (5.01-7.02)	<0.05
Sepsis	196,809 (15.8%)	418 (49.8%)	5.28 (4.61-6.05)	<0.0001	196,138 (15.2%)	286 (43.3%)	4.27 (3.66-4.98)	<0.05
Cardiopulmonary arrest	28,649 (2.3%)	218 (26.0%)	14.97 (12.82-17.47)	<0.0001	23,227 (1.8%)	129 (19.5%)	13.33 (11.00-16.17)	<0.05
Died during hospitalization	165,669 (13.3%)	470 (56.0%)	8.30 (7.24-9.51)	<0.0001	158,717 (12.3%)	256 (38.8%)	4.53 (3.88-5.30)	<0.05

**Table 5:** In-hospital outcomes of ARDS patients with and without ECMO subgrouped by age. Multivariable logistic regression was used to adjust for demographics (age, sex, race), hospital characteristics (region, bed size, teaching status), and all comorbidities listed in Table 1. The allowable tolerance for the underlying logistic regression model was set at 0.02 unit difference in odds of “between neighboring pairs.” ARDS: acute respiratory distress syndrome; ECMO: extracorporeal membrane oxygenation; aOR: adjusted odds ratio; AKI: acute kidney injury.

	Age < 65 years				Age > 65 years			
Outcomes	ARDS (N = 1,426,415)	ARDS on ECMO (N = 1412)	aOR (95% CI)	P-value	ARDS (N = 1,112,434)	ARDS on ECMO (N = 89)	aOR (95%CI)	P-value
AKI	192,566 (13.5%)	696 (49.3%)	6.25 (5.63-6.94)	<0.0001	248,073 (22.3%)	79 (88.8%)	27.59 (14.29-53.26)	<0.05
Major bleed	78,452 (5.5%)	339 (24.0%)	5.440 (4.81-6.15)	<0.0001	93,444 (8.4%)	30 (33.7%)	5.52 (3.56-8.57)	<0.05
Sepsis	202,551 (14.2%)	644 (45.6%)	5.05 (4.55-5.61)	<0.0001	189,114 (17.0%)	60 (67.4%)	10.07 (6.46-15.69)	<0.05
Cardiopulmonary arrest	28,528 (2.0%)	347 (24.6%)	15.97 (14.14-18.04)	<0.0001	23,361 (2.1%)	0 (0.0)	0.000	>0.05
Died during hospitalization	121,245 (8.5%)	603 (42.7%)	9.62 (8.66-10.68)	<0.0001	202,463 (18.2%)	60 (67.4%)	9.27 (5.95-14.45)	<0.05

## Discussion

Our study examined 2,540,350 patients with ARDS from 2002 to 2017. We report the following findings: (1) within the study period, ARDS with ECMO was associated with higher in-hospital mortality, cardiopulmonary arrest, major bleeding, AKI, and higher hospital stay and cost. (2) Our results demonstrated that regardless of age or sex, treating ARDS with ECMO resulted in significantly worse outcomes. (3) When examining VV or VA ECMO versus no ECMO in ARDS, patients treated with VV ECMO had significantly lower odds of in-hospital mortality and cardiopulmonary arrest.

ARDS is a life-threatening lung injury, ranging from mild to severe presentations. It is associated with significant morbidity and mortality with an estimated 40% in-hospital mortality [1]. Furthermore, it has been shown that increased disease severity is associated with an increased chance of mortality [18]. Despite significant advances in ventilatory therapy, prone positioning, and neuromuscular blockade, patients with severe ARDS may continue to decompensate and be placed on ECMO. The choice to initiate ECMO on a patient with ARDS is usually determined by multiple factors, including severe hypoxemia (partial pressure of oxygen/fraction of inspired oxygen < 80%), uncompensated hypercapnia with acidemia (pH < 7.15), or excessively high end-inspiratory plateau pressures (>35-45 cmH2O) despite low volume, and low-pressure ventilation [19].

Although originally believed that it would provide mortality benefits for patients, the efficacy of ECMO in severe ARDS patients remains controversial [3]. In a landmark study performed in the 1970s, Zapol et al. reported that ECMO did not increase the probability of long-term survival in patients with acute respiratory failure [20]. In a more recent study, the EOLIA trial examined treatment options for a group of patients with severe ARDS. It was shown that among patients with severe ARDS, 60-day mortality did not differ between a group treated with ECMO and a group treated with conventional mechanical ventilation therapy that included ECMO as rescue therapy [3]. Furthermore, the frequency of complications did not significantly differ between the ECMO group and the control group, except for more bleeding events requiring transfusion and severe thrombocytopenia in the ECMO group [3]. The results published in these studies were consistent with our current findings. The potential predictors of mortality in our analysis encompass patient age, comorbidities, and complications during ECMO therapy. Furthermore, previous studies have shown that, compared to shorter duration, prolonged ECMO support has been associated with decreased survival [21-23]. Although mortality benefits have been sparse and ECMO has resulted in deleterious events, Grant et al. argued that the benefits from this treatment may be dependent on the ventilator settings itself in which a true lung protective strategy must be adopted [24]. On the contrary, in the meta-analysis by Combes et al., the authors reported a lower 90-day mortality in patients with ARDS treated with ECMO compared to conventional treatment methods [8]. Furthermore, ECMO is thought to improve survival in trauma patients with severe post-traumatic ARDS [25].

Despite the fact that it was once believed that ECMO resulted in severe consequences for patients, more recent literature demonstrated improved outcomes in ARDS patients treated specifically with VV ECMO [26,27]. In the CESAR study, a multicenter randomized controlled trial, the use of ECMO significantly improved survival without severe disability in adult patients with severe but potentially reversible respiratory failure compared to conventional ventilation support [26]. In 2017, Kon et al. found that in patients with ARDS requiring pre-ECMO and/or inotropic support, VV ECMO may be initially instituted, reserving VA ECMO for conversion of uncontrollable hypotension. This resulted in significantly increased survival to discharge when compared to VA ECMO, and could even be utilized as an independent predictor of survival to discharge. VV ECMO was not associated with worse complication rates when compared to VA ECMO [28].

While we did not directly compare VV to VA ECMO, when VV ECMO was compared to no ECMO, we demonstrated consistent findings with previous literature supporting decreased mortality and minimal complications when comparing VA vs. no ECMO. Our results were also consistent with a recent retrospective report on 40 patients, which found that 85% of the patients on VV ECMO with severe ARDS from COVID-19 not only were able to be extubated early but were still alive. Also, 73% of patients survived hospital discharge with no oxygen requirement. Furthermore, complications were minimal and there were no ischemic strokes, inotropic support, or need for tracheostomy due to the early extubating strategy [29].

Currently, there is no objective data to explain the reason for the potential positive outcomes of VV ECMO in patients with ARDS [3]. It seems plausible that these findings may be because VV ECMO simply provides support to the lungs and prevents the development of cor-pulmonale. Ventilator settings and possible reduced workload on the lungs could also play a role.

Previous literature seems to support the idea that improved patient outcomes may not be directly due to VV ECMO but also the continuous advances in technology and the improved management of these critically ill patients [24,29].

There were multiple limitations in this study. The NIS is a large, nationally representative database that has been validated multiple times for accuracy. Nevertheless, as with all studies that use routinely collected electronic healthcare data, our research has several limitations. Given the study's retrospective design, the possibility of unmeasured confounding is present due to the lack of randomization. This analysis relied on ICD-9-CM codes, which may contain errors in the coding of diseases or procedures. There was also an absence of important information such as physical examination findings, medications, and laboratory results to determine the severity of ARDS. Furthermore, the analysis was only limited to in-hospital outcomes as follow-up after discharge was not available.

## Conclusions

In conclusion, the use of ECMO was associated with a significant increase in in-hospital outcomes in patients with ARDS. Furthermore, our findings add support to recent literature reporting decreased mortality and complications associated with VV ECMO and demonstrate a possible emerging therapeutic option for ARDS patients. Further work is needed to elucidate why VV ECMO has started to become a beneficial treatment for patients with ARDS.

## Appendices

Table S1

**Table 6:** The list of ICD-CM codes used in the analysis. ARDS: acute respiratory distress syndrome; ECMO: extracorporeal membrane oxygenation; VV: venovenous; VA: venoarterial; ICD-CM: International Classification of Diseases Clinical Modification; ICD-9: International Classification of Diseases, Ninth Revision; ICD-10: International Classification of Diseases, Tenth Revision.

Condition	ICD-9 code	ICD-10 code
ARDS	518.81	J80.XX
ECMO	39.65	
VV ECMO		5A1522H
VA ECMO		5A1522G

Table S2

**Table 7:** List of potential confounders accounted for regression analysis. ECMO: extracorporeal membrane oxygenation; VV: venovenous; VA: venoarterial.

ECMO
VA ECMO
VV ECMO
Peptic ulcer disease
Pulmonary disease
Pulmonary vascular disease
Alcohol use
Anemia
Electrolyte abnormalities
Hypothyroidism
Weight loss
Obesity
Paralysis
Depression
Coagulopathy
Peripheral artery disease
Family history of premature coronary artery disease
Obstructive sleep apnea
Liver disease
Drug use
Smoking
Prior percutaneous coronary intervention
Prior coronary artery bypass graft surgery
Heart failure
Prior myocardial infarction
Atrial fibrillation
Atrial flutter
Aortic stenosis
Mitral stenosis
Coronary artery disease
End-stage renal disease
Dyslipidemia
Hypertension
Diabetes mellitus
